# Facilitative Effects of *Alnus viridis* ssp. *fruticosa* on *Betula nana* ssp. *exilis* Growth in Arctic Alaska

**DOI:** 10.1002/ece3.73535

**Published:** 2026-06-10

**Authors:** Jackson W. Drew, Marion Syndonia Bret‐Harte, Agata Buchwal, Roger Ruess

**Affiliations:** ^1^ Department of Biology and Wildlife University of Alaska Fairbanks Fairbanks Alaska USA; ^2^ Institute of Arctic Biology University of Alaska Fairbanks Fairbanks Alaska USA; ^3^ Laboratory of Tree‐Ring Research University of Arizona Tucson Arizona USA; ^4^ Institute of Geoecology and Geoinformation Adam Mickiewicz University Poznan Poland

**Keywords:** *Alnus* and *Betula*, arctic plant ecology, climate change, competition, dendrochronology, facilitation, plant interactions, tundra shrubs

## Abstract

Arctic deciduous shrubs have responded to warming by increasing cover and extent, but how plant interactions (i.e., facilitation and competition) among deciduous shrub species might influence their climate sensitivities and overall growth remains unclear. Here, we investigate two deciduous shrubs: *Betula nana* ssp. *exilis* (dwarf birch) and *Alnus viridis* ssp. *fruticosa* (Siberian alder), the Arctic's only tall N‐fixing shrub, to determine how Siberian alder affects the growth of nearby dwarf birch. We harvested dwarf birch shrubs growing ‘near’ (i.e., within 1 m) and ‘away’ (≥ 3 m) from Siberian alders growing along the Sagwon Bluffs in Arctic Alaska for dendrochronological analysis. Dwarf birch traits and other site characteristics, including height, leaf N, catkin production, frost damage, and snow depth, were measured at additional sites nearby. We discovered locations ‘near’ Siberian alder had greater snow depth, which delayed the growing season for nearby dwarf birch, but likely protected them from early‐season frost damage. We estimated that radial growth occurring within 1979–2016 was delayed by 9 days, and peak radial growth was delayed by 2 weeks, for dwarf birch growing ‘near’ as compared to ‘away’ from alder. This resulted in different climate sensitivities for dwarf birch between locations. Measures of dwarf birch height, catkin abundance, leaf N, and average growth ring area were greater for plants ‘near’ alder than those ‘away’ from alder. This suggests that Siberian alder increased N mineralization and soil N availability under the deeper snowpack. Siberian alder facilitated dwarf birch growth. Through plant–plant interactions, Siberian alder altered the strength and type of stressors dwarf birch experienced, resulting in a change to the seasonality of its radial growth. Therefore, high‐spatial resolution species composition data could be included in vegetation models to predict future tundra growth responses more accurately to a changing climate.

## Graphical abstract

We investigated how Siberian alder influences the growth of dwarf birch in the low Alaskan Arctic. Using growth rings, we found that Siberian alder promotes dwarf birch growth overall despite delaying and shortening the growing season through enhancing snow depths.

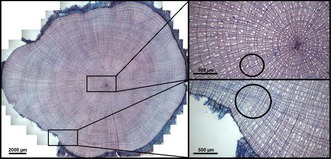

## 1 Introduction

The Arctic is among the most rapidly warming regions on Earth, where mean annual air temperatures have risen on average by 3°C over the period of 1971–2019 (AMAP 2021). In response to climate warming, plant communities are reorganizing. For example, deciduous shrubs in the Arctic have increased in abundance over the past several decades (Sturm, Racine, and Tape 2001; Tape et al. 2012; Buchwal et al. 2020; Myers‐Smith et al. 2020). Two co‐occurring deciduous shrubs that are rapidly and positively responding to ambient temperature increase in the Arctic are *Betula nana ssp. exilis* , hereafter dwarf birch, and *Alnus viridis ssp. fruticosa* , hereafter Siberian alder (Sturm, Racine, and Tape 2001; Tape et al. 2012; Cameron and Lantz 2016; Frost and Epstein 2014; Drew et al. 2023). Dwarf birch is distributed widely across the low Arctic, whereas Siberian alder has a more limited distribution, but is important for ecosystem N cycling because it is the only large (often > 1 m tall) N‐fixing shrub found in the Arctic (Tape et al. 2012; Cameron and Lantz 2016; Frost and Epstein 2014).

Facilitative and competitive plant interactions are likely to change in the warming Arctic, and these interactions will depend upon the types of stressors that plants encounter, and the relative strength of each type of interaction (Bertness and Callaway 1994), as well as the life history traits of the plants involved (Maestre et al. 2009). The stress gradient hypothesis (SGH) predicts more facilitation under high abiotic stress, and more competition under low abiotic stress (Bertness and Callaway 1994). A refinement of the SGH predicts that two competitive plants, as defined by (Philip 1977), can have net facilitative interactions if the primary stressor is resource‐based and moderate (Maestre et al. 2009). Importantly, interactions may not shift monotonically along stress gradients when multiple resources are limited; alleviation of one constraint (e.g., nitrogen) can intensify competition for others (e.g., light or phosphorus), resulting in spatially and temporally variable net interactions.

Under nutrient‐limited environments such as the Arctic (Chapin and Shaver 1985; Kremers et al. 2015), warming during winter may increase turnover of soil organic matter (SOM), and hence, soil nutrient availability (Schimel et al. 2004; Buckeridge and Grogan 2008), which may shift interactions toward facilitation under nitrogen limitation, but could also intensify competition for other resources between deciduous shrubs as they move from high to moderate resource‐based stress (Maestre et al. 2009). A higher abundance of deciduous shrubs, with their relatively decomposable leaf litter (Díaz et al. 2016), would tend to stimulate decomposition and thus increase nutrient availability overall. By examining the influences of Siberian alder on dwarf birch growth (i.e., net facilitative or competitive), we can infer whether the spread of dwarf birch will be enhanced or slowed down by alder in similar environments across the Arctic. Considering the circum‐arctic dwarf birch distribution, facilitation of birch growth will have important tundra ecosystem‐level feedback.

Alder can alter nutrient dynamics (Salmon et al. 2019) in the Arctic through its N‐rich leaf litter and through promoting greater overwinter mineralization from SOM by trapping wind‐blown snow (DeMarco et al. 2011; Sturm, Holmgren, et al. 2001), which may facilitate growth of nearby plants. Fertilization experiments have been shown to increase radial growth and height of deciduous shrubs, including dwarf birch and *Salix* spp. (Bret‐Harte et al. 2002; Campioli et al. 2012), which may facilitate shifts in community composition. Community composition shifts induced by experimental fertilization (Bret‐Harte et al. 2008; Chapin III et al. 1995) appear to favor deciduous over evergreen shrubs that may better be able to take advantage of increased N availability (Chapin III and Shaver 1996; Díaz et al. 2016), though this depends on the strength of fertilization applied (Campioli et al. 2012). In addition, greater snow depths promoted by alder may facilitate growth of nearby plants by providing greater moisture availability in spring and protection from early‐season freeze–thaw events (Hollesen et al. 2015), which are expected to increase in frequency (Landrum and Holland 2020).

Alder can also impose strong competitive effects on nearby plants by reducing both soil phosphorus (P) and light availability, and by delaying phenology associated with increased snow accumulation. To sustain N‐fixation, alder requires large amounts of P (Ruess et al. 2013), which may reduce P availability to other plants. In addition, alder is tall for an arctic shrub because it is heavily defended chemically against herbivores (Bryant et al. 1983) and grows in dense thickets, which shade shorter nearby plants (Craine and Dybzinski 2013), including dwarf birch (which is normally < 1 m tall). As the Arctic warms, understory plants are expected to suffer the most because of light competition with taller shrubs (Mekonnen et al. 2018) if tall shrubs become more abundant. Greater snow depths can delay the start of the dwarf birch growing season (Borner et al. 2008; Torp et al. 2010; Wilcox et al. 2019; Power et al. 2024), and thus deeper snow depths induced by Siberian alder may do the same. This could further reduce dwarf birch growth, especially if P and light are also limiting. Together, these mechanisms suggest that alder may simultaneously alleviate nitrogen limitation while intensifying limitation of light, phosphorus, and growing season length, leading to net interaction outcomes that depend on local resource balance and environmental context. Therefore, the net effect of alder on dwarf birch growth is not expected to be uniformly facilitative or competitive, but instead to vary across space and time depending on the relative strength of these opposing mechanisms.

Plant interactions are usually assessed on short time scales, using short‐lived plants. Dendrochronology enables assessment of woody plant growth and climate sensitivity in long lived arctic woody shrubs (Myers‐Smith et al. 2015; Buchwal et al. 2020). Long‐term analysis of growth rings from shrubs adjacent to alders can be used to detect different climatic sensitivities compared to shrubs growing away from alders over time. In addition, radial growth can be used to infer whether two shrub species' relationship is net facilitative or competitive over the long term (García‐Cervigón 2016; Liancourt and Doleźal 2023; Malfasi and Cannone 2020) by comparing average growth ring widths between members of the same species growing near and away from the other species. However, to our knowledge, this has not been done in the Arctic (Power et al. 2022). Given alder's potential to induce local scale changes in nutrients (Ruess et al. 2013, 2019; Salmon et al. 2019), hydrology, and snow capture (Sturm, Holmgren, et al. 2001), interaction effects are likely to be detected through dwarf birch radial growth and can help determine the mechanisms by which alder impacts dwarf birch growth, which is one of the most studied tundra shrub across the Arctic (Blok et al. 2010; Bret‐Harte et al. 2002; Buchwal et al. 2023; Gamm et al. 2018; Hollesen et al. 2015; Young et al. 2016; Zamin and Grogan 2012).

Here, we used dendrochronology to investigate the influence of Siberian alder on long‐term dwarf birch growth. We hypothesized that the radial growth of dwarf birch growing away from alder is more sensitive to June air temperatures than the dwarf birch growing near alder, where prolonged snow cover may delay the start of the growing season, resulting in greater sensitivity to July and August temperatures. Finally, we hypothesized that dwarf birch receives a net long‐term facilitative benefit growing near alder because of higher available soil N from decomposition of N‐rich alder leaf litter and greater overwinter mineralization.

## 2 Methods

### 2.1 Study Site

Study sites are in the Alaskan Arctic, USA, along south‐east facing toe slopes of the Sagwon Bluffs and in the floodplain of the Sagavanirktok River (69°26′35″ N 148°30′10″ W; Alaska Department of Natural Resources permit number LAS 31907; Drew et al. 2023). Approximately 100 km south of Prudhoe Bay, these sites lie within the low shrub Arctic zone (Walker et al. 2002). Average July and January air temperatures were 11.2°C and −27.3°C, respectively, for the period 1901–2016 (Climatic Research Unit version 4.03, Harris et al. 2020). Soil temperature was measured at 5 cm depth at 10 paired sites located ‘near’ (within 1 m) and ‘away’ (≥ 10 m) from alder between June 29, 2017, and June 24, 2018, using two iButton data loggers (iButtonLink, Whitewater, WI) per site and location. Soil moisture was measured at the same paired sites on July 7, 2018, using a HydroSense Soil Water Measurement System (Campbell Scientific Inc., Logan, UT). Additional soil cores were collected on August 15, 2018, to determine gravimetric soil moisture, and thaw depth was measured at the same time. Shrub sites ranged in elevation from 195 to 260 m a.s.l. (mean = 207 ± 17 m, *n* = 23) and were distributed along 1.5 km of the Sagwon Bluffs. Vegetation includes heath tundra at the tops of the bluffs and moist acidic tundra along the toe slope below the bluffs. The vegetation cover is mostly composed of deciduous shrubs ( *A. viridis* ssp. *fruticosa* , *B. nana* ssp. *exilis* , *Salix* spp.), graminoids (*Carex* spp. and *Eriophorum vaginatum* ), ericaceous shrubs (*Vaccinium* spp., *Rhododendron tomentosum*, and *Empetrum nigrum* ), mosses (*Aulacomnium* spp. and *Hylocomnium splendens*), and lichens, with forbs (*Lupinus arcticus, Petasites frigidus*, *Pedicularis* spp., *Stelarria longipes*, and *Pyrola asarifolia* ) occurring intermittently. Sites were selected to have common overstory shrubs (alder, dwarf birch, *Salix* spp., and *Rhododendron tomentosum* ). Alder dominated the sites, growing both as individual shrubs and in larger patches. Dwarf birch is one of the most abundant shrubs growing interspersed between and adjacent to alder shrubs or patches.

### 2.2 Shrub Sampling

To understand how climate sensitivity and long‐term radial growth of dwarf birch are influenced by proximity to alder, we harvested 23 dwarf birch growing ‘near’ (within 1 m) and 23 dwarf birch growing ‘away’ (≥ 3 m, usually 10 m) from alder shrubs or patches (Drew et al. 2023) over the years 2017–2018. Distance from alder shrubs was selected to ensure that sampled dwarf birch individuals were sufficiently separated to minimize alder‐associated nutrient influences (Salmon et al. 2019), while still maintaining an adequate pool of dwarf birch candidates for sampling. To determine differences in growth between ‘near’ and ‘away’ dwarf birch caused by the alder, shrubs were selected to be free of obvious disease. Dwarf birch shrubs were excavated to retrieve as much of the belowground structure as possible, including the main root.

To accurately date birch shrubs, we used a serial sectioning technique (Kolishchuk 1990). A minimum of three stem cross‐sections were cut from each shrub, ≥ 5 cm apart, to capture the variability of the shrubs' radial growth across the shrub. To estimate absolute shrub age, at least one above‐ and one belowground structure (i.e., stem, root, or collar) were sampled from an individual shrub. In total, up to nine cross‐sections were sampled from an individual birch shrub. The number of total cross‐sections taken per shrub depended on the need to resolve growth rings, resulting in 84 ‘near’ and 90 ‘away’ dwarf birch cross‐sections from the 46 individual shrubs analyzed.

**FIGURE 1:**
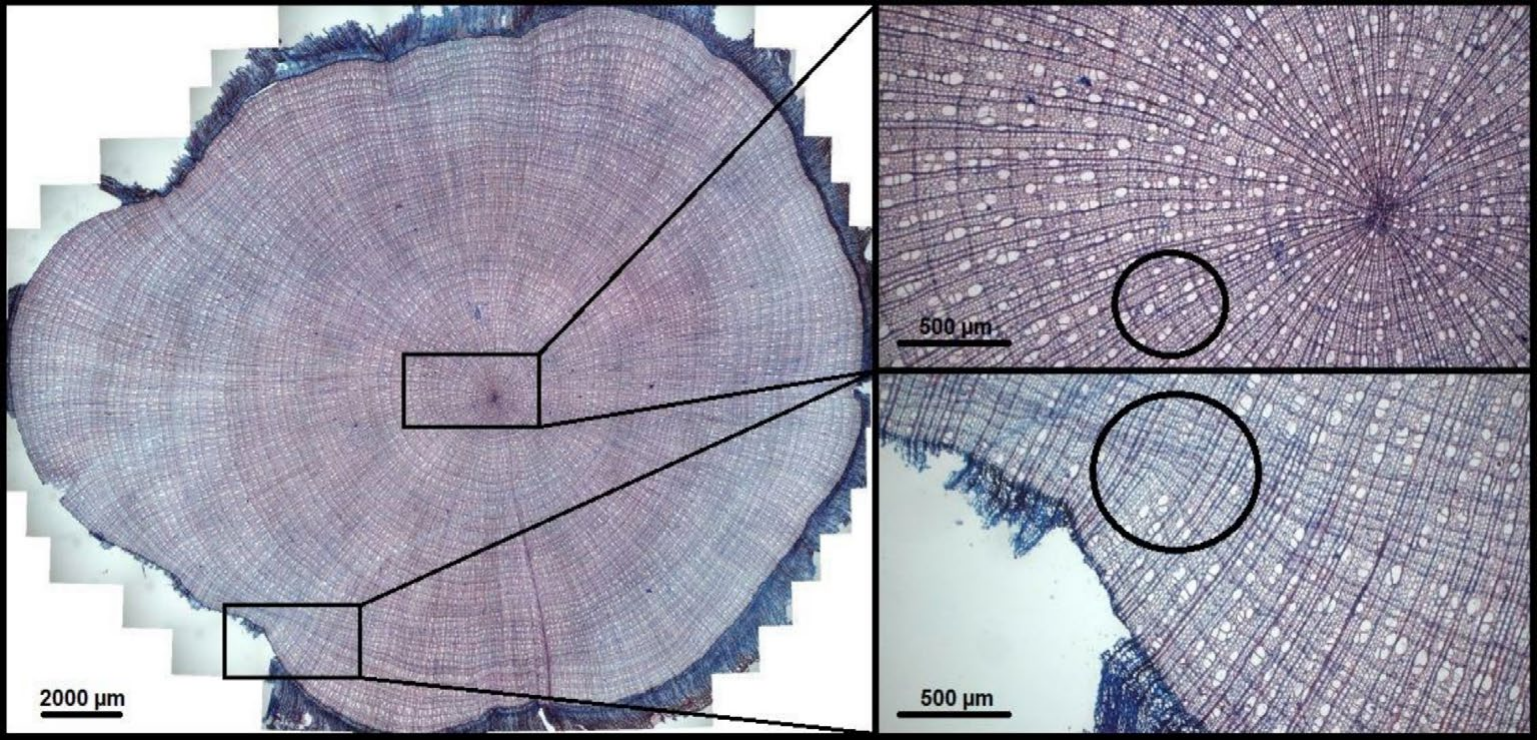
Composite image (left panel) of a *Betula nana* ssp. *exilis* root cross‐section. Wedging rings (black circles) are common and can occur early (top right) and later (bottom right) in their growth.

To analyze radial growth of dwarf birch, thin sections (approximately 20 μm thick) were cut from shrub cross‐sections using a GLS‐1 sledge microtome (WSL, Zurich; Gärtner et al. 2014). These thin sections were then stained with a 1:2 mixture of 1% aqueous solution of Safranin (Ward's Natural Science, Rochester) and Astra blue (Marker Gene Technologies, Eugene), washed with 95% ethanol, and fixed onto microscope slides with Canada balsam (Gärtner et al. 2014). Photographs of each thin‐section were taken using a digital camera (Excellis HDS 600, Accu‐Scope Inc., Commack) attached to a compound microscope (Leica DME, Leica Camera AG, Wetzlar) under 40× magnification and were merged into a single image (Figure 1) using Adobe Photoshop CS6 (Adobe Systems Incorporated, San Jose).

### 2.3 Cross‐Dating

The widths of annual growth rings were measured to the micron (0.001 mm) using ImageJ version 1.52p (Rasband 1998). We measured widths on a minimum of four radii per cross‐section to capture the variability of growth in each stem cross‐section (Buras and Wilmking 2014) and to enhance detection of wedging rings (Figure 1). Using positive and negative pointer years (i.e., years of high or low growth), we aligned the time series for each of the radii in a single cross‐section to resolve problematic rings and to ensure the accuracy of the growth series produced from each cross‐section (Schweingruber et al. 1990). Missing rings were given a value of one micron (Drew et al. 2023). Missing rings made up 3.7% and 3.6% of all rings from the ‘away’ and ‘near’ chronologies, respectively. Missing rings are a known limitation in Arctic shrub dendrochronology; however, their low frequency (3.6%–3.7%) and near identical occurrence between ‘near’ and ‘away’ chronologies suggest minimal risk of systematic bias in our comparisons. Consequently, although some uncertainty in absolute growth estimates may remain, relative differences between treatments are unlikely to be affected. Time series were then aligned between cross‐sections of a single shrub, and mean shrub time‐series were cross‐dated between the individual shrubs, to obtain the final mean time‐series (Myers‐Smith et al. 2015). Using COFECHA version 6.06 (Holmes 1983), cross‐dating quality was checked, and descriptive statistics were calculated.

### 2.4 Detrending

Basal area increment (BAI) was calculated from measured ring widths to account for geometric effects associated with increasing stem size (Johnson and Abrams 2009). Although BAI reduces size‐related growth trends, no additional detrending (e.g., curve fitting or spline detrending) was applied, as our objective was to retain low‐frequency variability potentially associated with ecological interactions (e.g., competition through P limitations or facilitation through N additions) between alder and dwarf birch. Although the BAI series may retain residual low‐frequency trends, in our system, these are interpreted as biologically meaningful signals of ecological interaction rather than artifacts requiring removal. Cross‐section BAI growth curves were pre‐whitened and then averaged at the shrub level for all 46 shrubs. The resulting shrub growth curves were averaged using a bi‐robust mean to create a stand chronology for both the ‘near’ (*n* = 23) and ‘away’ (*n* = 23) population of dwarf birch. The strength of the common signal was calculated for raw, BAI‐transformed, and pre‐whitened BAI chronologies, for both ‘near’ and ‘away’ populations, using the mean correlation between shrubs (Rbt) (Briffa and Jones 1990) and the expressed population signal (EPS) (Wigley et al. 1984). Growth series and chronology statistics were generated using the dplR package version 1.7.4 (Bunn et al. 2022) in R (version 4.3.0, R Core Team 2023). All subsequent analyses of growth rings used the pre‐whitened BAI chronologies.

**Table:** 

Replication statement			
Experiment	Scale of inference	Scale at which the factor of interest is applied	Number of replicates at the appropriate scale
Climate sensitivity	Plant	‘Near’ or ‘away’ alder	Time series of BAI spanning 1947–2016 or 1979–2016 composed of 23 individuals each
Dwarf birch traits and snow depth	Plant/site	‘Near’ or ‘away’ alder	12 of each combination
Average height and BAI	Plant	‘Near’ or ‘away’ alder	23 of each combination

### 2.5 Climate‐Growth Relationships

Using the treeclim package version 2.0.6.0 (Zang and Biondi 2015) in R, bootstrapped correlations (at 1000 iterations with replacement) between shrub growth and monthly average air temperature and precipitation were computed for both ‘near’ and ‘away’ chronologies (Climatic Research Unit version 4.03, Harris et al. 2020). Because of a significant positive trend in the temperature series, a cubic spline (at 2/3rd length of series) was applied to each monthly air temperature time‐series to remove long‐term trends and retain high frequency variation (Trouillier et al. 2019; Drew et al. 2023). Additionally, bootstrapped moving correlation functions (MCF) were performed between the ‘near’ and ‘away’ chronologies and both monthly temperature and precipitation. The window length in the MCF was set for 20 years, with a one‐year lag. All correlations were made for the period from 1947 to 2016 to ensure that the sample depth of each chronology included at least five shrubs throughout the entire period.

To explore the importance of daily temperatures on shrub growth during different periods within the growing season, we used the dendroTools package version 1.2.8 (Jevšenak and Levanič 2018) in R. We fit linear models with a fixed moving window length of 20 days, bootstrapped 1000 times with replacement, using daily average air temperatures for both ‘near’ and ‘away’ stand chronologies to find the part of the growing season that explained the most variation in radial growth of dwarf birch. A 20‐day window length was chosen to enable finer identification of the critical growing period without becoming spurious through too small a window. Daily average air temperatures for the period of 1979–2016 were acquired from OpenWeather (retrieved on November 3rd, 2021). OpenWeather's summer air temperature data had no significant trend, thus it was not detrended.

### 2.6 Sampling Dwarf Birch Traits and Snow Depths

To determine how alder influences the growth and habitat conditions of dwarf birch, we investigated 12 locations separate from where shrubs were harvested. At these 12 locations, between June 22 and 27, 2019, we measured the height of the tallest dwarf birch stem, collected green leaves, counted the number of buds damaged by frost, and the number of catkins from five different branches of dwarf birch ‘near’ and ‘away’ from alder on the north and south side of the alder. After collection, leaves were dried at 60°C for at least 3 days, ground to a fine powder with a mortar and pestle, and analyzed for N content using a CHN Analyzer (LECO CN628, LECO Corporation, St. Joseph, MI). In addition, we measured late spring snow depths on April 15, 2021, and April 25, 2022, from the 12 ‘near’ and ‘away’ locations on both the north and south side of the alder.

### 2.7 Analyses of Dwarf Birch Traits

We fit two‐way analysis of variance (ANOVA; *α* < 0.05) models to determine whether proximity to alder (i.e., ‘near’ vs. ‘away’), and relative aspect (i.e., north vs. south) to the alder canopy affected height and leaf nitrogen concentration of dwarf birch. The relative aspect was considered because overwinter wind direction can influence snow depths, which in turn may affect overwinter mineralization by altering soil temperature. In addition, we fit a one‐way ANOVA to determine whether dwarf birch height and average BAI differed between ‘near’ and ‘away’ shrubs whose rings were analyzed. Tukey post hoc tests were used to determine significant differences between groups when the overall ANOVA was significant.

Dwarf birch proximity to alder may influence the number of catkins produced, the frequency of any catkin production by individual shrubs, and the density of catkins (number of catkins/cm of stem analyzed) from shrubs that produced catkins, we fit three general linear models (GLM). To assess differences in the number of catkins produced, we fit a GLM with a negative binomial distribution using the ‘MASS’ package version 7.3.58.4 (Venables and Ripley 2002). To test the chance that dwarf birch produced catkins (frequency), we fit a GLM using a binomial distribution. Lastly, we modeled the non‐zero data using the “glmmTMB” package version 1.1.9 (Brooks et al. 2017) with the beta distribution to determine if proximity to alder changed the density of catkins on stems. Alternative approaches using mixed models to address issues of non‐independence were explored for all previous models; however, the results were all qualitatively similar, thus, they are not presented.

To determine if proximity to alder reduced the proportion of buds damaged by frost because of potentially greater snow depths ‘near’ alder, we fit a generalized linear mixed model (GLMM) using the package ‘glmmTMB’ with a binomial distribution. ‘Site’ was included as a random intercept for the GLMM. Pseudo R‐squared values were derived using the ‘MuMIn’ package version 1.47.5 (Bartoń 2023).

### 2.8 Analysis of Snow Depth

To determine whether proximity to alder (i.e., ‘near’ vs. ‘away’) and relative aspect (i.e., north vs. south) impacted maximal snow depth, we fit two‐way analysis of variance (ANOVA; *α* < 0.05) models for each year separately (i.e., 2021 and 2022). Random effect by ‘Site’ was considered but did not improve the model fit, so it was not included. All other assumptions were met. Differences between groups were assessed using the Tukey post hoc test.

## 3 Results

### 3.1 Chronology Characteristics

**FIGURE 2:**
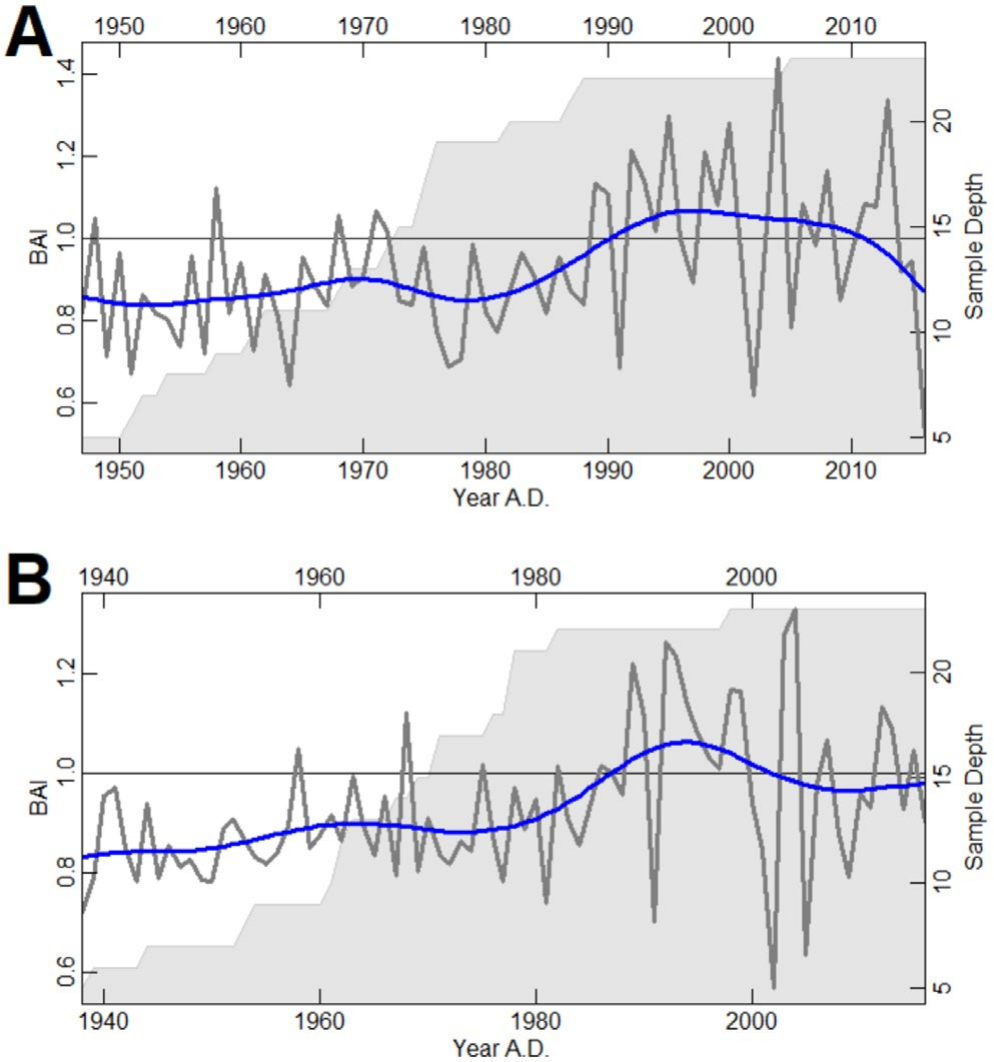
Time series of the pre‐whitened basal area increments (BAI, bold gray line) of (A) dwarf birch growing ‘away’ from alder (1947–2016) and (B) dwarf birch growing ‘near’ alder (1938–2016), with sample depth (number of samples at any point in time) indicated in gray shading. The blue line is the cubic spline (2/3rd length of series) applied to the chronology and is only used here for visual purposes.

Stand chronologies of dwarf birch growing ‘near’ alder spanned from 1938 to 2016, whereas those growing ‘away’ from alder spanned 1947 to 2016 (Figure 2). The mean age of ‘near’ dwarf birch was 61.2 ± 4.6 years, and for ‘away’ dwarf birch, it was 53.9 ± 3.6 years, which was not significantly different. The mean ring area of ‘near’ shrubs was significantly higher (1.55 ± 0.23 mm^2^/year) than for ‘away’ shrubs (1.01 ± 0.09 mm^2^/year.; *F*_1,44_ = 4.75; *p* = 0.035) for the common period of 1947–2016. The mean inter‐series correlation for the BAI‐transformed ‘near’ dwarf birch chronology was 0.279, whereas it was 0.315 for the ‘away’ dwarf birch chronology. The EPS for pre‐whitened ‘near’ and ‘away’ dwarf birch chronologies were 0.845 and 0.842, respectively (Table 1).

**TABLE 1:** Descriptive statistics for raw (i.e., ring width increment) and detrended ‘near’ and ‘away’ dwarf birch chronologies for the period 1938–2016 and 1947–2016, respectively. Raw chronologies were transformed using BAI. The near chronology was composed of 84 cross‐sections representing 23 shrubs, and the away chronology was composed of 90 cross‐sections representing 23 shrubs. The ‘rbt’ is the mean correlation between shrubs, and the ‘rwt’ is the mean correlation within shrubs. Expressed Population Signal (EPS) estimates how well a chronology on the basis of a limited number of shrubs represents the true chronology.

	Raw ‘Near’ chronology	BAI ‘Near’ chronology	Pre‐whitened BAI ‘Near’ chronology	Raw ‘Away’ chronology	BAI ‘Away’ chronology	Pre‐whitened BAI ‘Away’ chronology
rbt	0.174	0.315	0.199	0.189	0.336	0.203
rwt	0.294	0.279	0.144	0.364	0.315	0.231
EPS	0.823	0.910	0.845	0.837	0.918	0.842
Signal to noise ratio	4.64	10.11	5.46	5.14	11.14	5.34

### 3.2 Climatic Sensitivity of ‘Near’ and ‘Away’ Dwarf Birch Chronologies

**FIGURE 3:**
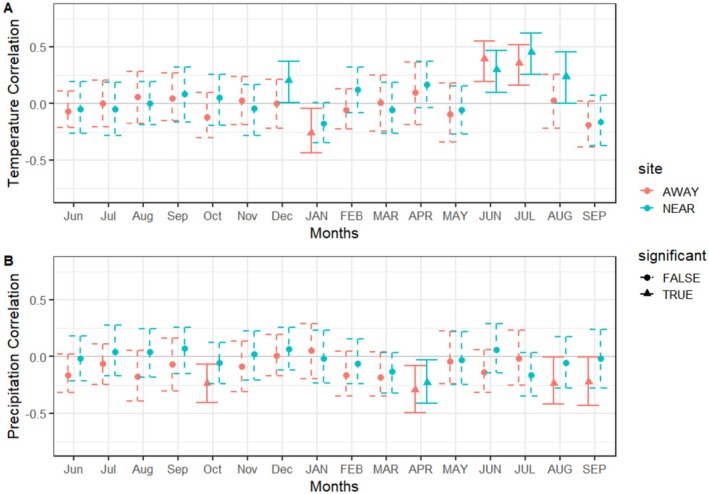
To examine how the ‘near’ (in blue) and ‘away’ (in red) dwarf birch chronologies correlated with (A) average monthly air temperature and (B) average monthly precipitation over the period of 1947–2016, we bootstrapped (1000 iterations) Pearson correlations of pre‐whitened basal area increments (BAI). Months in all capitals represent the current year's months, whereas months not in all capitals represent the previous year's months. Bars represent 95% confidence intervals. Significant (*α* = 0.05) correlations are represented by solid lines with triangles.

Radial growth of both dwarf birch chronologies was positively correlated with the current year's June and July mean air temperatures (Figure 3A). Growth of ‘near’ dwarf birch was positively correlated to the current year's August mean air temperature, whereas that of ‘away’ dwarf birch was not. In recent decades, both chronologies became increasingly positively correlated to the current year's June air temperatures, while, in contrast, less correlated to the current year's August air temperature (Figure S2).

The ‘away’ dwarf birch chronology was negatively correlated with precipitation in the previous year's October, and in the current year's April, August, and September (Figure 3B). In contrast, the ‘near’ dwarf birch chronology was negatively correlated with only the current year's April precipitation (Figure 3B). Inconsistent correlations between growth and precipitation were revealed in both chronologies, but recent decades showed that ‘near’ shrubs were increasingly negatively correlated with precipitation in the current year's February, April, and July (Figure S3).

### 3.3 High Resolution Climate Sensitivity

**FIGURE 4:**
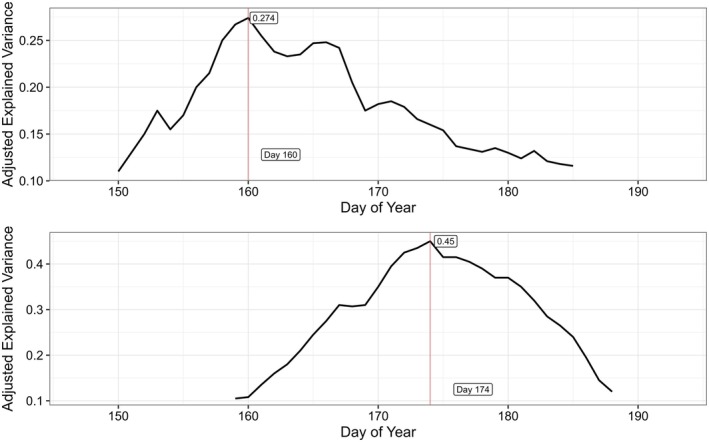
Results for bootstrapped (1000 iterations with replacement) linear models of (A) the ‘away’ and (B) the ‘near’ dwarf birch chronologies fit to daily average temperature (OpenWeather data) for the period of 1979–2016 with a fixed window length of 20 days. The red line indicates the beginning of the 20‐day window that explains the most variance. The black line indicates significant (*α* = 0.05) windows related to radial growth.

To explore the sensitivity of radial growth to air temperature within the growing season, we fit linear models of dwarf birch growth to daily average air temperatures. This analysis showed that the ‘near’ dwarf chronology had more variance explained relative to the ‘away’ birch chronology, for a fixed window length of 20 days (*R* ^2^ = 0.45 for ‘near’ dwarf birch; *R* ^2^ = 0.27 for ‘away’ dwarf birch; Figure 4). The timing of the 20‐day window that explained the most variance was notably different for birch growing in ‘near’ and ‘away’ locations: June 23rd–July 12th for ‘near’ birches, compared to June 9th–28th for ‘away’ birches. Models of ‘near’ birch radial growth were significant to a shorter (30 days) and later (day of year = 159–189) period of the summer than ‘away’ birch (36 days; day of year = 150–186) consistent with a delayed start and shorter growing season, potentially associated with deeper snowpack at ‘near’ sites. Average air temperature was generally higher in the later window than in the early season (Figure S4).

### 3.4 Differences Between Birch Traits and Snow Depth From ‘Near’ and ‘Away’ Locations

**FIGURE 5:**
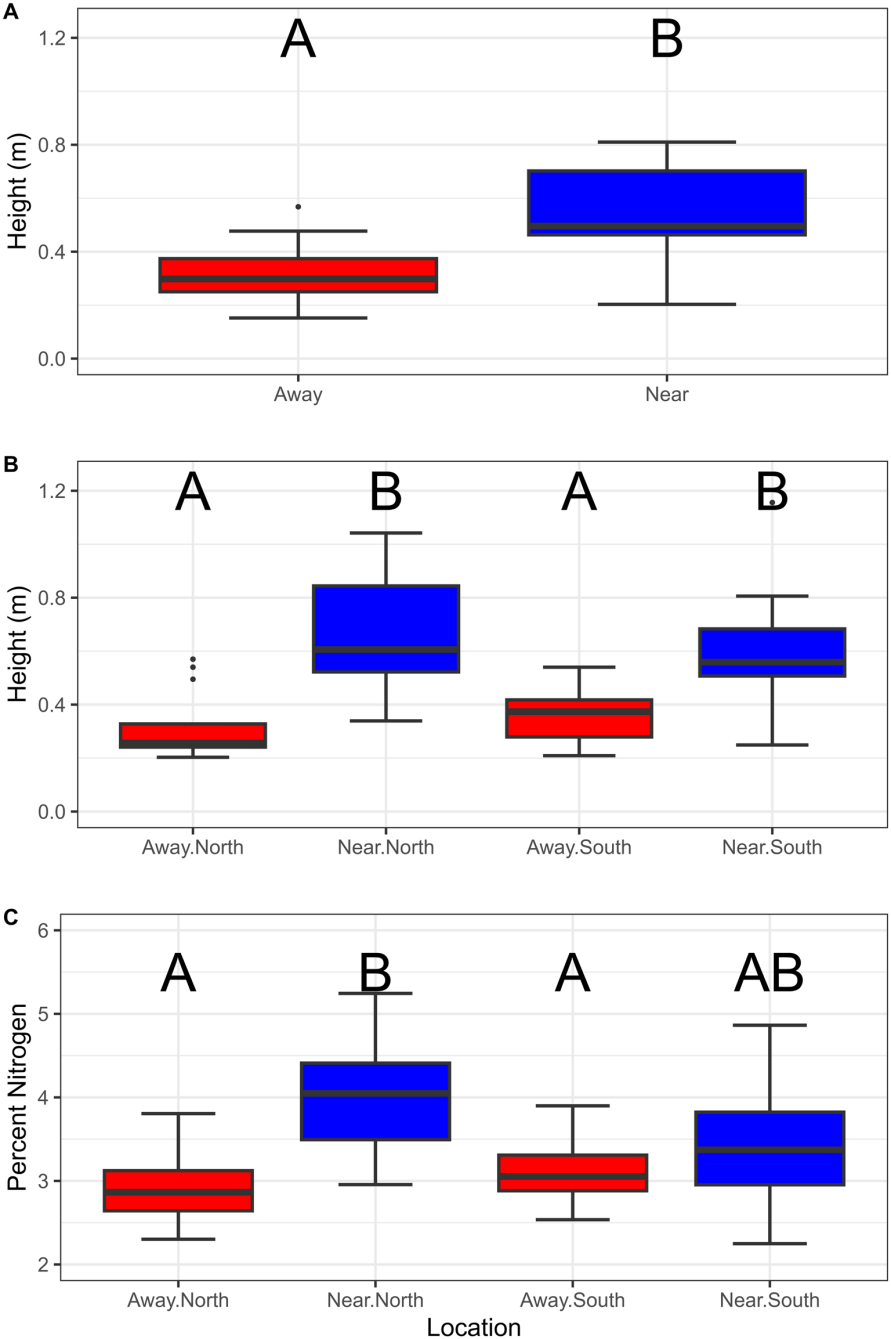
Box plots of (A) dwarf birch height by location (whose secondary rings were measured), (B) height of dwarf birch, and (C) leaf nitrogen by location and relative aspect to the alder (whose rings were not measured). Red boxes represent birch sites ‘away’ from alder, and blue boxes are birch sites ‘near’ alder. Box plots show the outliers (points), range (dotted lines), upper and lower quartiles (box), and the mean (bold line). Letters denote significant differences between groups *α* < 0.05.

Dwarf birch growing ‘near’ alder were significantly taller than those growing ‘away’ from alder (Figure 5A,B; Table 2A,B). Dwarf birch growing ‘near’ alder on the north side of the alder had significantly greater leaf nitrogen (4.0% N) than dwarf birch growing ‘away’ (North = 2.9% N; South = 3.1% N; n.s.) from alder (Figure 5C; Table 2C). Leaf nitrogen from dwarf birch growing on the south side ‘near’ alder was intermediate (3.4% N) between leaf nitrogen from dwarf birch growing ‘near’ alder on the north side and from dwarf birch ‘away’ from alder (Figure 5C). Some of the differences seen in percent leaf N content between ‘near’ and ‘away’ sites may reflect leaves that had not fully expanded at the time that leaves were collected in late June, because of thicker snow depths having delayed phenology (Torp et al. 2010) in the ‘near’ sites versus the ‘away’ sites.

**TABLE 2:** One‐way analysis of variance (ANOVA) model of (A) height of dwarf birch (whose rings were measured) as a function of proximity to alder (‘near’ vs. ‘away’ sites). Two‐way ANOVA model of (B) dwarf birch height and (C) dwarf birch percent leaf nitrogen (whose rings were not measured) as a function of proximity to alder interacting with aspect (north vs. south). Bold values indicate significance at *α* = 0.05.

S	Effect	DF	*F* value	*p*	*R* ^2^
A. Height	Location	1; 40	23.32	**< 0.0001**	0.37
B. Height	Aspect	1; 44	< 0.01	0.959	
	Location	1; 44	33.56	**< 0.0001**	0.44
	Aspect*Location	1; 44	1.04	0.314	
C. Leaf nitrogen	Aspect	1; 44	1.15	0.290	
	Location	1; 44	15.75	**< 0.001**	0.33
	Aspect*Location	1; 44	4.28	**0.044**	

**FIGURE 6:**
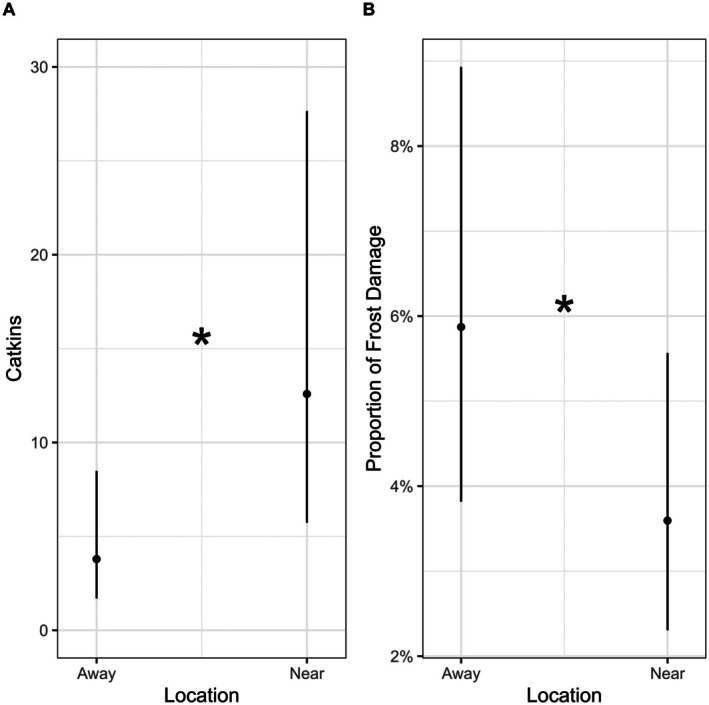
(A) The number of catkins on the basis of location (i.e., ‘away’ or ‘near’ site from alder) and (B) the proportion of buds damaged by frost on the basis of location. Bars represent 95% confidence intervals, and ‘*’ indicates the groups are significantly different (*α* = 0.05).

**FIGURE 7:**
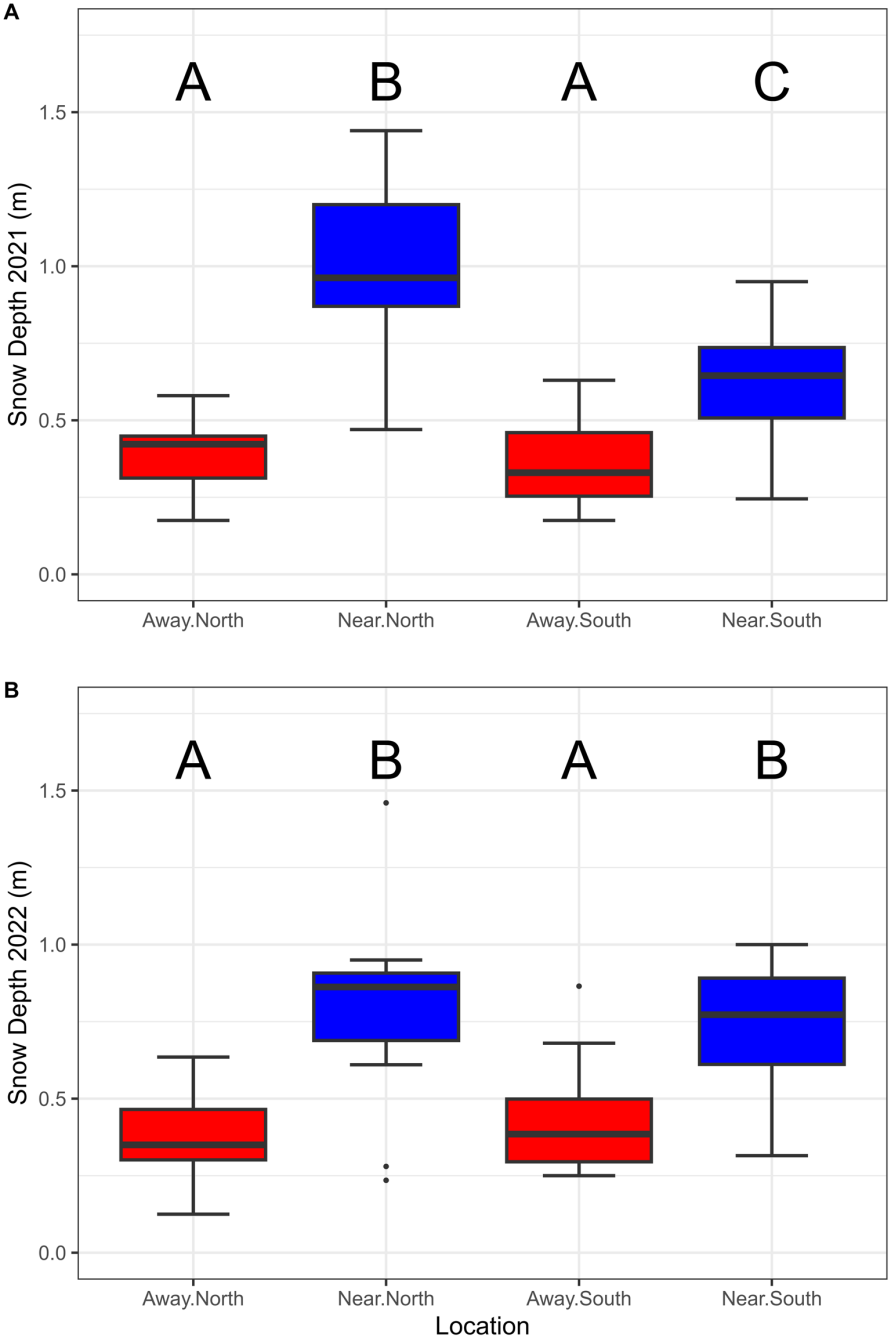
Box plots of snow depth on the north and south side, near (blue) and away (red) from the alder for the year 2021 (A) and 2022 (B), respectively. Box plots show the outliers (points), range (bars), upper and lower quartiles (box), and the mean (bold line). Letters denote significant (*α* < 0.05) differences between groups.

Dwarf birch growing ‘near’ alder had more catkins overall than dwarf birch growing ‘away’ from alder (Figure 6A, Table 3A), but neither the chance of producing catkins (*X* ^2^_1,45_ = 0.37; *p* = 0.544) nor catkin density (*X* ^2^_1,24_ = 0.66; *p* = 0.417) differed between locations. However, these non‐significant differences should be interpreted with caution, as variability among individuals may obscure subtle differences between groups. Dwarf birch growing ‘near’ alder was less prone to frost damage than dwarf birch growing ‘away’ from alder (Figure 6B, Table 3B). Late winter snow depth was twice as deep at ‘near’ sites as at ‘away’ sites (0.78 ± 0.04 m for ‘near’ and 0.39 ± 0.02 m for ‘away’; averaged for both N and S sites across both years) (Figure 7; Table 4). Soil temperatures were slightly warmer at ‘near’ sites during winter (January–April; +0.68°C) and slightly cooler during the remainder of the year (−0.44°C; Figure S1). Thaw depth tended to be shallower at ‘near’ sites (41.7 cm) compared to ‘away’ sites (55.7 cm; df = 9, *t* = 2.16, *p* = 0.06). Soil moisture was lower at ‘near’ sites in July (4.26% vs. 16.7%; df = 9, *t* = 2.00, *p* = 0.08), but similar between groups later in the growing season (August: 332% vs. 387%; df = 9, *t* = 0.75, *p* = 0.47).

**TABLE 3:** Analysis of deviance tables for models relating proximity to alder (i.e., ‘away’ vs. ‘near’ sites) to (A) number of catkins and (B) proportion of frost damage. The number of catkins was modeled with a general linear model using a negative binomial model. The proportion of frost damage was modeled with a generalized linear mixed model using a binomial distribution. ‘Site’ is included as a random effect for the ‘frost’ model. Bold values indicate significance at *α* = 0.05. Pseudo *R* ^2^ was calculated using the ‘delta’ method.

	Effect	DF	*X* ^2^	*p*	*R* ^2^	adj *R* ^2^
A. Catkin model	Location	1; 46	4.14	**0.042**	0.08	0.08

	Effect	DF	*X* ^2^	*p*	Marginal *R* ^2^	Conditional *R* ^2^
B Frost model	Location	1; 45	15.11	**< 0.001**	0.09	0.76

**TABLE 4:** Two‐way analysis of variance (ANOVA) models of (A) snow depth in year 2021 and (B) snow depth in 2022 as a function of proximity to alder (‘near’ vs. ‘away’ sites) interacting with aspect (north vs. south). Bold values indicate significance at *α* = 0.05.

	Effect	DF	*F* value	*p*	*R* ^2^
Snow depth 2021	Aspect	1; 44	12.50	**0.001**	
	Location	1; 44	58.96	**< 0.0001**	0.65
	Aspect*Location	1; 44	10.44	**0.002**	
Snow depth 2022	Aspect	1; 44	0.02	0.90	
	Location	1; 44	27.91	**< 0.0001**	0.39
	Aspect*Location	1; 44	0.76	0.39	

## 4 Discussion

Using dendrochronology, we were able to assess Siberian alder's effect on dwarf birch growth over a 70‐year period. This multi‐decadal annual growth comparison contrasts with most studies investigating plant—plant interactions, which usually evaluate relationships between short‐lived plants and/or on shorter‐term time scales (García‐Cervigón 2016). Moreover, in the Arctic plant—plant interactions have not been assessed previously using annual growth rings (Power et al. 2022). Analyzing radial growth of dwarf birch growing ‘near’ and ‘away’ from alder revealed differences in the timing and sensitivity of growth to air temperature. We estimate alder delayed the start of ‘near’ dwarf birch radial growth by 9 days and peak radial growth by 2 weeks (Figure 4). Changes in the timing of growth rings were probably driven by enhanced snow depths. Alder likely promoted nearby soil N availability through both direct and indirect effects. Overall enhanced N may have influenced ‘near’ dwarf birch climate sensitivity when temperatures were warmer, extending peak wood production into the growing season. Despite delays to the growing season, dwarf birches growing ‘near’ alder were taller, had greater ring area, number of catkins, leaf N, and less frost damage compared to those growing ‘away,’ indicating that alder facilitates dwarf birch growth over the long term.

### 4.1 Alder Promotes Dwarf Birch Growth

Alder may improve soil N availability directly through the decomposition of its N‐rich leaf litter, which has been shown to be important at other sites adjacent to alder in the Arctic (Salmon et al. 2019; Drew et al. 2023). Indirectly, alder could increase soil N availability by trapping and accumulating wind‐blown snow. Deeper snow insulates soils, promoting overwinter mineralization (Sturm, Holmgren, et al. 2001), which likely promotes increased radial growth of arctic shrubs, including dwarf birch (DeMarco et al. 2011, 2014; Addis and Bret‐Harte 2019; Campioli et al. 2012; Bret‐Harte et al. 2002; Prager et al. 2020).

As the Arctic continues to warm, abiotic stressors such as strong winds and early season freeze–thaw events are projected to increase in frequency (Landrum and Holland 2020). Dwarf birch growing near alder with deeper snow would be less prone to incur damage during winter and springtime when such events are most likely to inflict damage to plants (Figure 6B). Protection from physical stress has benefitted dwarf birch and may become more beneficial over time if resource stress lessens (Maestre et al. 2009). Up to this point, facilitation by alder appears to have outweighed competitive effects at spatial and temporal scales examined here because dwarf birch growing near alder was taller, with greater ring area, more catkins, and less frost damage than those growing away from alder, despite being of similar age.

However, competitive interactions may become more pronounced over longer temporal scales or at different spatial scales than those examined here. Increased N availability associated with alder may also intensify competition for other limiting resources such as P or light, potentially leading to delayed or scale‐dependent competitive effects. Consequently, these results should be interpreted as context‐dependent, with the balance between facilitation and competition likely varying across environmental gradients and stages of shrub development.

### 4.2 Differences in Phenology

Increased snow depths likely delayed leaf‐out and radial growth of ‘near’ dwarf birch (Borner et al. 2008; Torp et al. 2010). Despite this, soil temperatures during the growing season were similar between ‘near’ and ‘away’ sites (Figure S1). This likely reflects the insulating effect of snow during winter and the rapid increase in soil temperature following snowmelt, such that differences in the timing of snow disappearance are not well captured by growing season soil temperature measurements. As a result, soil temperature records may not reflect early‐season phenological delays driven by deeper snowpack.

Comparable Siberian alder sites in the western Canadian Arctic (Wilcox et al. 2019) had deeper, denser snow than *Betula glandulosa* sites, which were similar in snow depths, latitudes, and aspects to our sites, and led to an approximate 2‐week delay in snow melt. A snow fence experiment across multiple habitat types in Abisko, Sweden, consistently showed differences in snow melt date that resulted in much smaller differences to dwarf birch leaf out (Torp et al. 2010), supporting our estimated nine‐day delay in radial growth due to snow addition. Delayed phenology may also influence trait measurements; for example, higher leaf nitrogen observed at ‘near’ sites could partially reflect incomplete leaf expansion at the time of sampling, as leaves developing under deeper snowpack may have been at an earlier phenological stage (Torp et al. 2010).

The timing of peak wood formation was likely influenced by snow additions as well, but was delayed further because having enhanced N at this site enabled more growth during the warmer part of the growing season. This is supported by the much greater amount of variance explained in ‘near’ dwarf birch as compared to ‘away’ dwarf birch. As the growing season lengthens in the Arctic because of climate change (Jeong et al. 2011), this implies that nutrient availability can modulate radial growth seasonality, possibly lengthening or shortening the period during which radial growth is sensitive to climate.

### 4.3 Ecological Consequences

In response to Arctic warming, deciduous shrubs, including dwarf birch (Bret‐Harte et al. 2008) and alder (Drew et al. 2023), have increased their growth in many arctic locations (Myers‐Smith et al. 2020; Buchwal et al. 2020), making it necessary to better understand plant interactions between shrub species. We found that Siberian alder facilitates dwarf birch growth by reducing both ambient resource stress and physical stress (Maestre et al. 2009), likely by enhancing both snow depths and soil N availability. Moreover, alder promoted dwarf birch growth and catkin production, suggesting that co‐occurrence of alder may also promote dwarf birch expansion by enhancing reproductive output. Increased dwarf birch abundance may also have broader ecological consequences. For example, expansion of deciduous shrubs can alter forage availability and quality for herbivores (Christie et al. 2015; Bryant et al. 2014) and may suppress understory forbs through increased shading. However, the extent and direction of these cascading effects are likely to vary across ecosystems and require further study.

Plant traits for both the benefactor and beneficiary species are known to heavily influence plant interaction outcomes (He et al. 2013). Siberian alder has two traits that ameliorate the environment for dwarf birch: its ability to improve local soil N availability and its large biomass that enables enhanced snow capture, which likely lowers physical stressors such as frost damage. Although Siberian alder is the only large N‐fixing shrub in the Arctic, there are other tall shrubs (> 1 m), such as *B. glandulosa* and *Salix* spp. (Myers‐Smith et al. 2015), which are getting taller with Arctic warming (Bjorkman et al. 2018) and may mimic these same mechanisms with other shrubs.

Despite these patterns, several limitations should be considered when interpreting our results. We acknowledge the potential for site selection bias, as areas where alder is established may inherently represent more favorable microsites (e.g., deeper soils, different moisture availability, or reduced exposure). In addition, this work does not include direct measures of soil nutrients, which may differ from previously reported studies (Salmon et al. 2019). Although our paired ‘near’ and ‘away’ design minimizes these effects at local scales, we cannot fully exclude the possibility that some observed differences reflect underlying environmental heterogeneity rather than alder presence alone.

## 5 Conclusion

We assessed the long‐term effects of Siberian alder on dwarf birch by quantifying radial growth responses of birch shrubs over a 70‐year period. To our knowledge, a dendrochronological approach has not been used within the Arctic to assess plant–plant interactions before. We found that Siberian alder promotes the growth of nearby dwarf birch shrubs, probably by reducing both abiotic and resource stress. Furthermore, Siberian alder alters dwarf birch climate sensitivity by delaying its radial growth, probably by enhancing snow depths, and possibly extends the timing of peak wood production because of enhanced N during warmer temperatures, which promoted greater growth. This shows alder's capacity to change long‐term local abiotic conditions to affect long‐term plant growth, even as larger synoptic patterns of warmer air temperature improved conditions for both shrubs over the study period at our site (Buchwal et al. 2020; Myers‐Smith et al. 2020; Tape et al. 2012; Cameron and Lantz 2016; Frost and Epstein 2014; Drew et al. 2023), and demonstrates important pathways by which shrubs may interact with each other in the future. The strength of these interactions will be dependent on the species traits involved (He et al. 2013) and their environment, so it will be necessary to model shrub‐species composition at high spatial resolution to accurately predict future tundra growth responses to climate change.

## Author Contributions

**Jackson W. Drew:** conceptualization (lead), data curation (lead), formal analysis (lead), investigation (lead), methodology (lead), visualization (lead), writing – original draft (lead), writing – review and editing (lead). **Marion Syndonia Bret‐Harte:** conceptualization (equal), funding acquisition (lead), investigation (supporting), methodology (supporting), project administration (equal), resources (lead), software (lead), supervision (lead), writing – original draft (supporting), writing – review and editing (equal). **Agata Buchwal:** conceptualization (equal), data curation (supporting), formal analysis (equal), investigation (equal), methodology (equal), supervision (equal), validation (equal), visualization (supporting), writing – original draft (supporting), writing – review and editing (equal). **Roger Ruess:** conceptualization (equal), funding acquisition (lead), investigation (supporting), project administration (equal), resources (lead), supervision (equal), visualization (supporting), writing – original draft (supporting), writing – review and editing (equal).

## Funding

This work was supported by the Office of Polar Programs, 1503912, 1623461, 1936752. Division of Environmental Biology, 1556481.

## Conflicts of Interest

The authors declare no conflicts of interest.

## Supporting information


**Figure S1:** Average daily soil temperature time‐series (measurements performed at 5 cm depth from June 29, 2017 to June 24, 2018 at 10 paired sites) representative for ‘near’ and ‘away’ dwarf birches sites.
**Figure S2:** Moving correlation functions of how the dwarf birch chronologies correlated with average air temperatures (1947–2016) to (A) ‘away’ locations and (B) ‘near’ locations. We bootstrapped (1000 iterations) Pearson correlations of pre‐whitened basal area increments (BAI). Window length for all correlations equals 20 years with one‐year offset. Significant (α = 0.05) correlations are represented by asterisks (*). Color represents direction and strength of correlation, where the darkest blue is highly positively correlated and the darkest red is highly negatively correlated.
**Figure S3:** Moving correlation functions of how the dwarf birch chronologies correlated with monthly precipitation (1947–2016) to (A) ‘away’ locations and (B) ‘near’ locations. We bootstrapped (1000 iterations) Pearson correlations of pre‐whitened basal area increments (BAI). Window length for all correlations equals 20 years with one‐year offset. Significant (α = 0.05) correlations are represented by asterisks (*). Color represents direction and strength of correlation, where the darkest blue is highly positively correlated and the darkest red is highly negatively correlated.
**Figure S4:** The average air temperature during the 20‐day period that explained the most variation in growth for dwarf birch growing ‘near’ (orange) compared to ‘away’ (blue) from alder. The window period for the ‘away’ dwarf birch spanned from June 9th to 28th, whereas for the ‘near’ dwarf birch, it spanned from June 23rd to July 12th. The timing of the window was determined by when the most variation of secondary growth was explained for each site.

## Data Availability

Data available from the Arctic Data Center: https://doi.org/10.18739/A27P8TG2F (Drew et al., 2026).
