# Supplementary material for: Facilitative Effects of Alnus viridis ssp. fruticosa on Betula nana ssp. exilis Growth in Arctic Alaska
Source: Ecol Evol. 2026 Jun 10;16(6):e73535. doi: 10.1002/ece3.73535 (PMC13250631; doi:10.1002/ece3.73535)
Supplement: Supplementary file 1 — Figure S1: Average daily soil temperature time‐series (measurements performed at 5 cm depth from June 29, 2017 to June 24, 2018 at 10 paired sites) representative for ‘near’ and ‘away’ dwarf birches sites. Figure S2: Moving correlation functions of how the dwarf birch chronologies correlated with average air temperatures (1947–2016) to (A) ‘away’ locations and (B) ‘near’ locations. We bootstrapped (1000 iterations) Pearson correlations of pre‐whitened basal area increments (BAI). Window length for all correlations equals 20 years with one‐year offset. Significant (α = 0.05) correlations are represented by asterisks (*). Color represents direction and strength of correlation, where the darkest blue is highly positively correlated and the darkest red is highly negatively correlated. Figure S3: Moving correlation functions of how the dwarf birch chronologies correlated with monthly precipitation (1947–2016) to (A) ‘away’ locations and (B) ‘near’ locations. We bootstrapped (1000 iterations) Pearson correlations of pre‐whitened basal area increments (BAI). Window length for all correlations equals 20 years with one‐year offset. Significant (α = 0.05) correlations are represented by asterisks (*). Color represents direction and strength of correlation, where the darkest blue is highly positively correlated and the darkest red is highly negatively correlated. Figure S4: The average air temperature during the 20‐day period that explained the most variation in growth for dwarf birch growing ‘near’ (orange) compared to ‘away’ (blue) from alder. The window period for the ‘away’ dwarf birch spanned from June 9th to 28th, whereas for the ‘near’ dwarf birch, it spanned from June 23rd to July 12th. The timing of the window was determined by when the most variation of secondary growth was explained for each site. [file ECE3-16-e73535-s001.docx]

Supplemental Figures

Figure S1

Average daily soil temperature time-series (measurements performed at 5 cm depth from June 29, 2017 to June 24, 2018 at ten paired sites) representative for ‘near’ and ‘away’ dwarf birches sites.


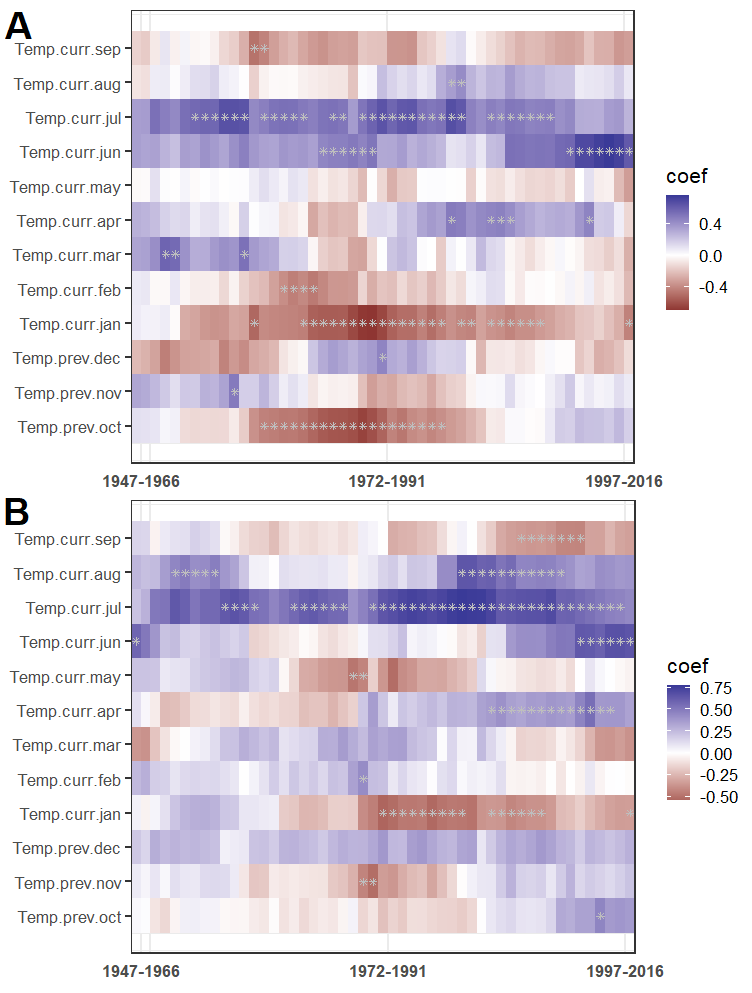


Figure S2

Moving correlation functions of how the dwarf birch chronologies correlated with average air temperatures (1947 – 2016) to A) ‘away’ locations and B) ‘near’ locations. We bootstrapped (1,000 iterations) Pearson correlations of pre-whitened basal area increments (BAI). Window length for all correlations equals 20 years with one-year offset. Significant (α = 0.05) correlations are represented by asterisks (*). Color represents direction and strength of correlation where darkest blue is highly positively correlated and darkest red is highly negatively correlated.


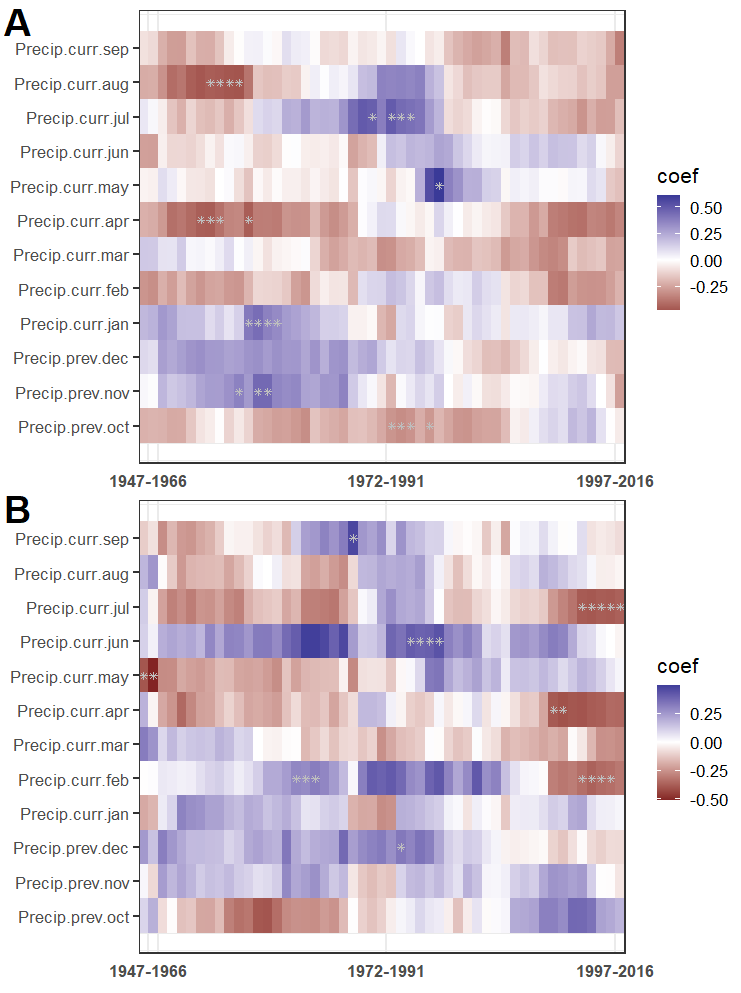


Figure S3

Moving correlation functions of how the dwarf birch chronologies correlated with monthly precipitation (1947 – 2016) to A) ‘away’ locations and B) ‘near’ locations. We bootstrapped (1,000 iterations) Pearson correlations of pre-whitened basal area increments (BAI). Window length for all correlations equals 20 years with one-year offset. Significant (α = 0.05) correlations are represented by asterisks (*). Color represents direction and strength of correlation where darkest blue is highly positively correlated and darkest red is highly negatively correlated.

:

Figure S4

The average air temperature during the 20-day period that explained the most variation in growth for dwarf birch growing ‘near’ (orange) compared to ‘away’ (blue) from alder. The window period for the ‘away’ dwarf birch spanned June 9^th^ – 28^th^, while for the ‘near’ dwarf birch it spanned from June 23^rd^ – July 12^th^. The timing of the window was determined by when the most variation of secondary growth was explained for each site.
